# Morphometric and Enzymatic Changes in Gills of Rainbow Trout after Exposure to Elevated Temperature—Indications for Gill Remodeling

**DOI:** 10.3390/ani14060919

**Published:** 2024-03-16

**Authors:** Franz Lahnsteiner

**Affiliations:** 1 Federal Agency for Water Management, Institute for Water Ecology, Fisheries and Lake Research, Scharfling 18, 5310 Mondsee, Austria; franz.lahnsteiner@baw.at; 2 Fishfarm Kreuzstein, Oberburgau 28, 4866 Unterach, Austria

**Keywords:** gill, elevated temperature, morphometry, enzymes, remodeling

## Abstract

**Simple Summary:**

When rainbow trout are exposed to elevated temperatures over the long term, adaptations occur. Understanding these adaptation processes can aid in evaluating the response of this fish species and related species to climate change, as well as in determining the maximum temperature they can tolerate before compromising their welfare. The present study aimed to investigate whether adaptations could also be observed in the gills. Gill structure and enzyme activities were examined in fish exposed to 20 °C for 32 days, in comparison to fish maintained at their temperature optimum of 9 °C. The study revealed significant changes in gill structure and metabolism, indicative of an adaptive response to elevated temperatures.

**Abstract:**

Seven-month-old rainbow trout acclimated to 9 °C were used. The fish were gradually adapted to a water temperature of 20 °C over a period of seven days and then exposed to this temperature for 32 days. Changes in gill morphometry and histology and in enzyme activities in comparison to fish kept at 9 °C were investigated. No histopathological abnormalities were discerned at the heightened temperature. The gill epithelium thickened by approximately 40%, suggesting an increase in the branchial diffusion barrier for ions, water, and gases. Concurrently, there was a significant decrease in the activities of gill H^+^-ATPase and Na^+^/K^+^-ATPase, indicative of a reduction in osmoregulation under elevated temperatures. Carbonic anhydrase activity exhibited an increase following the 32-day exposure to 20 °C, potentially mitigating the adverse effects of increased gill epithelium thickness on gaseous exchange. There were no indications of gill surface enlargement as the measurements of the length of the primary and secondary lamellae, as well as of the distances between them, were similar at 9 and 20 °C. The activities of the gill enzymes associated with glycolysis and the citric acid cycle displayed a varied response following the 32-day exposure of rainbow trout to 20 °C. Pyruvate kinase decreased, while lactate dehydrogenase increased, and malate dehydrogenase remained constant. This might suggest a decrease in the glycolytic rate, a greater reliance on anaerobic pathways at 20 °C compared to 9 °C, and the consistent efficiency of the citric acid cycle in the gills of rainbow trout in response to elevated temperatures. In summation, the data suggest a remodeling of rainbow trout gills in response to elevated temperatures, affecting both morphometric and metabolic aspects.

## 1. Introduction

The abrupt exposure of teleost fish species to sudden temperature changes leads to thermal shock reactions, while gradual exposure, combined with a sufficiently long exposure time, induces acclimatization reactions [1]. Knowledge about acclimatization processes is important in ecology and fish culture for defining the plasticity of teleost fish species to temperature changes. The acclimatization potential of economically valuable salmonid species is particularly interesting for solving the problem of stock survival arising from the heat sensitivity of these cold-preferring species.

The gill of teleost fish is the dominant site for gas exchange, osmoregulation, acid–base regulation, and the excretion of nitrogenous waste [2]. Reversible gill remodeling is a process that describes the plasticity of the gill tissue in response to alterations in the environment [3]. During this process, the structure of the gills adjusts to adapt to specific environmental changes. Environmental factors leading to gill remodeling include water oxygen concentrations, water chemistry, temperature, and environmental pollutants [2,3]. The gill structure is determined by its respiratory and osmo/ionoregulatory function [2,3,4]. A large functional surface area and low diffusion distance favor O_2_ uptake, whereas a low functional surface area and high diffusion distance favor osmo/iono-regulation, a concept termed the “osmorespiratory compromise” [2,3,4]. There are different mechanisms by which fish can change the functional branchial area and diffusion distance, involving different forms of changes in blood flow and changes in the gill structure.

Temperature-mediated structural changes in gills which can be considered as a gill remodeling process have been detected in several fish species. The most prominent examples are crucian carp (*Carassius carassius*) and goldfish (*Carassius auratus)*. These species produce an interlamellar cell mass that fills the space between adjacent gill lamellae under cold water temperatures [3,5]. The cell mass is absorbed at warm water temperatures to increase the respiratory surface area [3,5]. In the Hoven’s carp (*Leptobarbus hoevenii*), a 20-d lasting temperature increase for 4 °C over the optimum decreases the gill epithelial thickness and changes the size of the secondary lamellae [6]. In tilapia (*Oreochromis niloticus*), a 15-week long exposure to water temperatures 8 °C over the optimum decreases the width of the secondary lamellae and causes the swelling of their terminal areas [7]. In a grouper (*Epinephelus*) hybrid, gill secondary lamella thickness increases in the lower and upper suboptimal temperature range [8]. A cold acclimation from 25 °C to 5 °C increases the branchial water–blood barrier thickness of the freshwater eel (*Anguilla anguilla*) [9]. Contrary, in five different coral reef species (*Acanthochromis polyacanthus*, *Chromis atripectoralis*, *Pomacentrus moluccensis*, *Dascyllus melanurus,* and *Cheilodipterus quinquelineatus*), no changes in gill structure in relation to temperature were observed [10]. Further, the pathological alteration of gills was frequently observed at a suboptimal temperature (*Epinephelus* hybrid [8]; *Leptobarbus hoevenii* [6]; rohu, *Labeo rohita* [11]; barramundi, *Lates calcarifer* [12]; Caspian brown trout, *Salmo caspius* [13].

Although salmonid species belong to the most important cultured fish species worldwide, the effects of elevated temperatures on gill structure and gill remodeling have not been investigated so far in these species. This knowledge can help to evaluate the response of Salmonidae to climate change, and to determine the maximum temperature that species can tolerate before their welfare is compromised.

The present study was conducted on rainbow trout (*Oncorhynchus mykiss*). Fish acclimated to 9 °C were exposed to 20 °C for 32 d. The primary objective was to discern any pathological changes in the gills triggered by the rise in temperature, as well as to detect potential morphometric or cellular alterations within the gills, indicative of a remodeling and adaptation process in response to elevated temperatures. In addition, the study measured the key enzymes for various metabolic processes. These included the enzymes indicative of ion transport (Na^+^/K^+^ ATPase, H^+^ ATPase), CO_2_ transport (carbonic anhydrase), glycolysis (pyruvate kinase, lactate dehydrogenase), and the citric acid cycle (malate dehydrogenase). The aim of these investigations was to gain a deeper understanding of whether structural modifications were correlated with functional alterations in the gills and whether temperature-related alterations also concerned the metabolism.

## 2. Materials and Methods

### 2.1. Experimental Fish

Seven-month-old rainbow trout acclimated to 9 ± 1 °C during their whole lives and adapted to a natural photoperiod typical for the Northern Hemisphere were used for the experiments. They derived from the commercial production of the fish farm Kreuzstein and were reared in circular tanks under flow-through conditions (1 L/s) and at a stocking density of 3000 fish per tank (8 fish/L). At the onset of the experiments, 40 fish were used for size determination and the body mass was 34.5 ± 9.8 g and the total length was 13.7 ± 0.6 cm. The experiments were carried out in accordance with Austrian regulations governing animal welfare and protection and with the EU directive 2010/63/EU for animal experiments.

### 2.2. Fish Exposure to Elevated Temperatures

**Figure 1:**
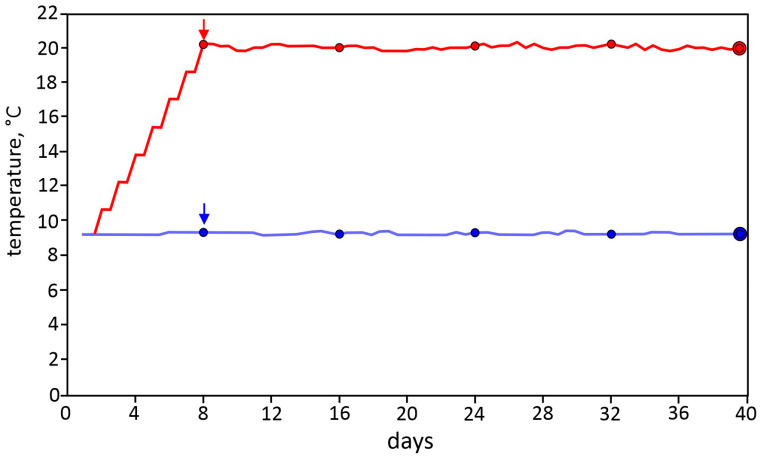
Temperature in the control (blue line) and temperature exposure (red line) stream channels. Data are the mean of the 2 replicates, standard deviation is not shown as it is <0.2 °C for all mean values. Red and blue arrows indicate start of experiment; small red and blue circles indicate date of fish sampling for mass determination; big red and blue circles indicate date of fish sampling for analysis.

Rainbow trout were exposed to 20 °C for 32 days. An exposure temperature of 20 °C was chosen because it is close to the critical thermal maximum of rainbow trout [14]. Thirty-two days were selected for exposure, as previous studies [15,16] demonstrated that this period is adequate to detect the long-term effects of temperature on the fish organism. Experiments were conducted in stream channels (190 × 25 × 35 cm, length × width × height) under flow-through conditions. Flow-through conditions are advantageous for this kind of experiment, as constant water conditions can be maintained throughout the experiment and tank effects are unlikely. The water supply was groundwater of constant quality, the flow rate was set to 0.2 L/s, and the stocked fish mass was 4 kg (110–120 fish) per stream channel. This experimental setup reflects conditions relevant to aquaculture. The total number of fish per stream channel was used for the determination of body mass and mortality. From the total number of fish per stream channel, a haphazard selection of fish was made for specific analysis procedures. Four stream channels were used. In two channels, the temperature was maintained at 9 °C and served as controls (Figure 1). The other two stream channels were gradually heated to 20 °C over a 7-day period using a geothermal heat pump (NibeF2300, Nibe AB, Markaryd, Sweden). The temperature was increased stepwise by 2.8 °C per day (Figure 1). The time point of temperature increase was 2:00–3:00 p.m.

When the water temperature had reached 20 °C, the experiment was started (Figure 1). The experiments were performed from 6 July to 7 August 2023, i.e., during the period when rainbow trout exhibited the highest tolerance to elevated temperatures [14]. The inspection and cleaning of stream channels were performed twice per day at 8:00 a.m. and 4:00 p.m., according to the hygienic concept of the fish farm. During the experiment, fish were fed a commercial trout diet at a ratio of 1.0% of the body weight over a period of 14 h per day using band feeders. Water parameters were investigated at regular intervals. The temperature was measured with Tinytag Aquatic 2 temperature loggers (Gemini Data Loggers Ltd., Chichester, UK), the oxygen concentration was measured with an optical sensor electrode FDO^®^ 925 (WTW, Xylem Analytics, Weilheim, Germany), the dissolved total gas pressure was measured with a saturometer GhPa-400 (Fish and Aqua Technology, Vilshofen, Germany), and NH_4_-N was measured with a colorimetric method [17].

The total fish mass per stream channel and the mass of 20 individual fish per stream channel were determined in 8-day intervals. Based on these data, the percentile daily increase in fish mass was determined (=daily growth rate). Dead fish were daily recorded for each tank. The survival rate was calculated as the number of fish counted in each stream channel at the end of the experiment in relation to the number of fish stocked at the onset of the experiment.

### 2.3. Sampling of Fish and Used Chemicals

At the end of the experiment, 10 fish per stream channel were haphazardly sampled and euthanized with 0.3% MS222. The total length and mass were determined. Blood was removed from the gills before sampling as erythrocyte enzyme activities might influence enzyme activity measurements in gill tissue. For this reason, the heart ventricle was opened to bleed the fish out. Then, the second gill arch was excised. It was selected as it is less affected by external factors than the first gill arch and it is also unlikely to be accidentally damaged during dissection. The gill arch of the right body side was used for morphometric investigations, and the gill arch of the left body side for enzymatic studies. For morphometric investigations, the gill arch was fixed in 0.1 mol/L of cacodylate buffered 4% glutaraldehyde solution (pH 7.4) at 4 °C. The tissue for enzymatic analysis was immersed in 100 mmol/L of Tris (pH 7.4), containing 5 mmol/L of EDTA, and 0.5% Triton X-100 and stored at −25 °C.

All chemicals were of analytical research grade and were obtained by Sigma-Aldrich (Merck KGaA, Vienna, Austria). Technovit 7100 was purchased by Kulzer GmbH (Werheim, Germany).

### 2.4. Histological Procedures

**Figure 2:**
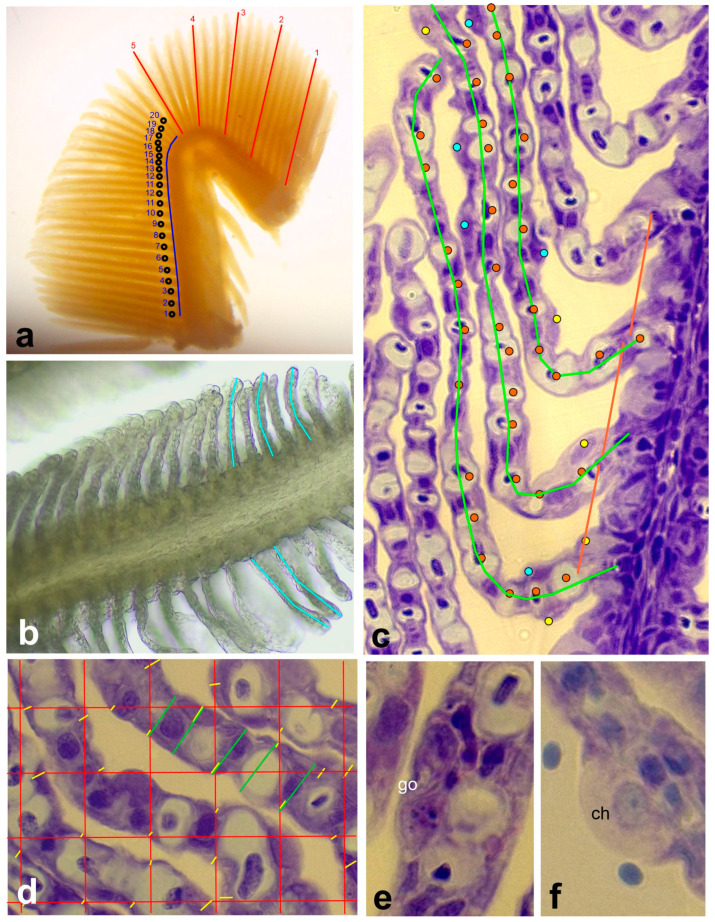
Morphometric landmarks measured in gills of rainbow trout. (**a**) Measurement of the length of primary lamella of the gill arch (red lines) and of the distance between primary lamellae. Twenty primary lamellae were counted and marked (black circles). The distance from the 1st to the 20th primary lamella was measured at the base of the gill arch (blue line) and divided by 20. Stereomicroscopic micrograph. (**b**) Measurement of length of secondary lamellae (blue line). Stereomicroscopic micrograph. (**c**) Measurement of the distance between secondary lamellae (red lines) and counts of mucus (blue circle) and chloride cells (yellow circle) and of capillaries (red circle). The secondary lamella was measured and the cell types and capillaries were counted and their number extrapolated to 1 mm. Histological section. (**d**) Measurement of secondary lamella and epithelium thickness. A grid was placed over the section and measurements were taken where the grid lines intersected the epithelium. Epithelial thickness measurements (yellow lines) are marked for the whole picture, secondary lamella measurements (green lines) only for 5 examples. Histological section. (**e**) Mucus cell (go). Histological section. (**f**) Chloride cell (ch). Histological section.

The fixed samples were photographed in a stereomicroscope at 10- to 25-fold magnification together with a calibration slide as shown in Figure 2a,b. Then, the samples were processed for histological analysis. After the fixative was rinsed out, the samples were decalcified in 10% EDTA solution, dehydrated in a graded series of ethanol, and embedded in Technovit 7100. Sections that were 3 μm in thickness were cut with a HM 325 Rotary Microtome (Thermo Fisher Scientific, Waltham, MA, USA). Sections from three different gill regions were prepared by the following cutting procedure: 5 sections were cut and sampled, and the following 25 sections were cut and discarded until 15 sections from three different regions were collected. Sections were stained with 0.1% toluidine blue diluted in 0.1 mol/L of phosphate buffer at pH 7.2.

### 2.5. Morphometric Analysis

All morphometric analyses were performed on micrographs in the Image J program. The length of the primary lamellae, the length of the secondary lamellae, and the distance between the primary lamellae were measured on micrographs taken in a Motic stereomicroscope DM-39C-N9GO with an integrated digital camera (Motic GmbH, Spilburg, Germany). The length of the primary lamellae and the distance between the primary lamellae were measured as shown in Figure 2a. The length was measured for every 5th primary lamella of the gill arch. To determine the distance between single primary lamellae, 20 primary lamellae were counted and marked. The distance from the 1st to the 20th primary lamella was measured at the base of the gill arch and divided by 20. Two measurements were taken per individual. The width of the primary lamellae was not measured as it is explained by the length of the secondary lamellae. The secondary lamellae were measured as shown in Figure 2b. Five measurements were performed on 5 different primary lamellae, respectively, resulting in a total of 25 measurements per individual.

The distance between the secondary lamellae, thickness of the epithelium, and number of capillaries, chloride cells, and mucus cells per mm secondary lamella were determined on histological sections in a Motic BA310 biological light microscope with a built-in 3.0 megapixel digital camera (Motic GmbH, Spilburg, Germany). Based on the applied cutting procedure, 5 histological sections were available from 3 different gill regions per individual. From each gill region, 1 high-quality section was selected and photographed at 400- and 1000-fold magnification (Figure 2c,d). Using the micrographs recorded at 400-fold magnification, the distance between the secondary lamellae was measured (Figure 2c). Eight measurements were made per section, yielding 24 measurements per individual. Then, the secondary lamella was measured and the cell types and capillaries were counted (Figure 2c) and extrapolated to 1 mm. The histological features of chloride cells and mucus cells are shown in Figure 2e,f. Finally, the height (i.e., the thickness) of the gill epithelium was measured on micrographs recorded at 1000-fold magnification. To obtain a random measurement design, a grid was placed over the section and measurements were taken where the grid lines intersected the epithelium. The measurement number was 15 per section and 45 per individual. For the different analyzed parameters, the mean per individual was calculated for further statistical analysis.

### 2.6. Histopathological Analysis

Gill histopathology was investigated on micrographs of histological sections of secondary lamellae recorded at 400-fold magnification: First, the number of fused and significantly curled secondary lamellae was counted per microscopic field and set in relation to the total number of secondary lamellae in the microscopic field. The determination was repeated 5 times per individual. Then, the length of the randomly selected secondary lamella was measured and the occurrence of the aneurysm of capillaries, of necrotic cells including cells with edema, and of sites with epithelial lifting was counted and extrapolated to a filament length of 1 mm. Eight measurements were made per section, resulting in 24 measurements per individual. Finally, the mean per individual was calculated for the histopathological alterations and used for further statistical analysis.

### 2.7. Enzymatic Assay

Gill tissue (100 ± 10 mg) suspended in 600 µL 100 mmol/L Tris (pH 7.4), containing 5 mmol/L EDTA, and 0.5% Triton X-100 was homogenized using a Dounce type tissue homogenizer (VWR International, Vienna, Austria) and centrifuged for 10 min at 1000× *g*, and the supernatant was used for analysis. This supernatant represents the crude enzyme extract of the sample. Protein concentration was determined in the crude enzyme extract and the enzyme activities were referred to the protein concentration of the samples (see below). The temperature of the enzymatic assays corresponded to the water temperature of the fish, i.e., 9 °C for control fish and 20 °C for fish exposed to elevated temperatures. Adequate blanks (no substrate, no sample) were used to run all enzymatic assays and specific inhibitors were used for Na^+^/K^+^ ATPase activity (2.5 mmol/L ouabain), H^+^-ATPase (50 µmol/L bafilomycin), and carbonic anhydrase (2 mmol/L acetazolamide) to exclude interactions from other enzymes in the crude extract. Optimal substrate and inhibitor concentrations were determined in preliminary experiments. Lactate dehydrogenase, pyruvate kinase, and malate dehydrogenase were assayed UV-spectrophotometrically [18]. For lactate dehydrogenase, the substrate was 2 mmol/L pyruvate and the assay was pH 7.4, for pyruvate kinase, the substrate was 3 mmol/L phosphoenolpyruvate and the assay was pH 7.8, and for malate dehydrogenase, the substrate was 2 mmol/L malate and the assay was pH 9.4. ATPases were determined by NADH-coupled enzymatic assays monitoring the amount of liberated ADP [19] with 5 mmol/L of ATP as the substrate in the presence of 100 mmol/L of NaCl and 20 mmol/L of KCl and at pH 7.6. Na^+^/K^+^ ATPase activity was measured in the presence and absence of 2.5 mmol/L of the specific inhibitor ouabain [20]. H^+^-ATPase was measured as the bafilomycin-sensitive ATPase activity in the presence and absence of 50 µmol/L of bafilomycin [20]. Carbonic anhydrase was determined colorimetrically by its esterase activity using 2 mmol/L 4-nitrophenyl acetate as a substrate and a sodium phosphate buffer of pH 7.5 supplemented with 300 mM of NaCl in the presence and absence of 2 mmol/L of the specific inhibitor acetazolamide [21]. Absorbance was measured with a Multiskan™ FC Microplate Photometer (Thermo Fisher Scientific, Waltham, MA, USA). Enzyme activities were referred to the protein concentration of the samples, which was determined with the Lowry procedure [22].

### 2.8. Statistics

JASP 0.14.1 software was used for statistical calculations. Before analysis, morphometric and enzymatic data were tested for normal distribution. The Shapiro–Wilk test was used to test for normal distribution as it is appropriate for small sample sizes (*n* < 50). This test revealed a normal distribution for all analyzed parameters. Then, the data were analyzed for potential tank effects. As no significant differences in the analytes’ values were detectable between the related tanks, datasets from the two 9 °C tanks and the two 20 °C tanks were pooled. Morphometric data were analyzed as non-transformed and as length-transformed datasets, as morphometric parameters might change with fish size. Transformation was performed according to the formula T_mp_ = MP × (M_TL_/I_TL_)^b^ (T_mp_: transformed parameter, MP: measured parameter, M_TL_: mean of fish total lengths, I_TL_: individual fish total length, b slope of the linear regression of the log of the measured parameter and the log of the transformed individual fish length) [23,24]. No differences in statistical analysis results were found between the two datasets. Non-transformed are presented in the results section. The Student’s *t*-test was used for statistical analysis at a probability level of *p* < 0.005.

## 3. Results

### 3.1. Water Parameters

The dissolved oxygen (O_2_) concentration was 11.59 ± 0.21 mg/L (*n* = 8) for water at 9 °C and 8.16 ± 0.25 mg/L for water at 20 °C, demonstrating a significant difference (*p* < 0.005). However, all other analyzed water parameters exhibited no difference between the two temperatures. Total gas saturation was 98.3 ± 1.5% (pooled data for 9 °C and 20 °C, n = 16), the pH was measured at 7.86 ± 0.01, conductivity was measured at 341.5 ± 8.0 µS/cm, acid-neutralizing capacities were measured at 3.35 ± 0.02 mval/L, while the PO_4_^3^- and NH_4_^+^ levels were 0.003 ± 0.001 mg/L and 0.003 ± 0.002 mg/L, respectively. Mn^2+^, Fe^2+^ or Fe^3+^, Zn^2+^, and Cu^2+^ were under the detection limit (<0.001 mg/L). Overall, all water parameters fell within the optimal range for rainbow trout [25].

### 3.2. Fish Parameters

There were no significant differences in fish mass between the two 9 °C stream channels and the two 20 °C stream channels at any investigation time point. However, fish growth was significantly (*p* < 0.005) higher at 9 °C compared to 20 °C. The total weight increase after 32 days was 128.1 ± 1.8% at 9 °C (*n* = 2, calculated for the two tank repetitions) and 109.7 ± 1.6% at 20 °C, with the daily growth rate of 4.0 ± 0.5% at 9 °C and 3.4 ± 0.4% at 20 °C. The survival rate of control fish was 98.3 ± 1.1% (two tank replicates), while that of the temperature-exposed fish was 97.8 ± 0.6%. The observed fish loss was due to manipulations for mass determination; however, no temperature-related mortality was recorded.

### 3.3. Gill Morphometry

The thickness of the gill epithelium and secondary lamellae significantly increased in rainbow trout exposed to 20 °C for 32 days compared to those acclimated to 9 °C (Table 1). However, parameters such as the length of the primary lamellae, distance between the primary lamellae, length of the secondary lamellae, distance between the secondary lamellae, as well as the number of mucus cells, chloride cells, and capillaries per mm secondary lamella remained unaffected by temperature changes (Table 1).

### 3.4. Histopathological Alterations of the Gills

No histopathological alterations were detectable in the gills of rainbow trout exposed to 20 °C for 32 days compared to those kept at 9 °C. The absence of fused or curled secondary lamellae, the aneurysms of capillaries, and the separation of the epithelium from the lamellae were noted. The number of necrotic cells in the gill epithelium was 5.2 ± 4.9/mm secondary lamella (*n* = 20) for fish kept at 9 °C and 6.5 ± 6.4/mm secondary lamella (*n* = 20) for fish kept at 20 °C.

### 3.5. Enzyme Activities

With the exception of malate dehydrogenase, all analyzed gill enzyme activities changed after exposure to elevated temperatures (Table 2). Lactate dehydrogenase and carbonic anhydrase activities increased, while pyruvate kinase, Na^+^/K^+^ ATPase, and H^+^-ATPase activities decreased (Table 2).

## 4. Discussion

Under the experimental conditions employed (slow temperature rise, low stocking density ensuring ample oxygen supply), rainbow trout exhibited adaptation to a temperature of 20 °C. No temperature-related mortalities were observed, and the fish demonstrated positive growth rates. Negative growth rates typically indicate significant cellular and endocrine stress, as cortisol reduces appetite and nutrient digestibility [26,27,28,29]. However, at 20 °C, the growth rate was lower than at 9 °C, suggesting a reallocation of energy resources from somatic growth to maintaining homeostasis [28,29]. Total gas saturation, pH, conductivity, acid-neutralizing capacities, and PO_4_^3−^, NH_4_^+^, Mn^2+^, Fe^2+^, Fe^3+^, Zn^2+^, and Cu^2+^ concentrations did not differ between 9 °C and 20 °C and were within the optimal range for rainbow trout. At 20 °C, the oxygen concentration of water was lower than at 9 °C, but still in the optimal range for rainbow trout. Contrarily, the water temperature of 20 °C was out of the optimal range [30] and close to the critical thermal maximum [14]. Therefore, temperature appears to be the sole environmental variable affecting the organism.

One significant morphometric alteration observed in rainbow trout exposed to 20 °C for 32 days was an increase in the thickness of the epithelium. This alteration was particularly notable, with a 40% increase compared to the 9 °C control. Additionally, the thickness of the secondary lamellae also increased. These changes are likely a consequence of the heightened epithelial dimension. No indications of gill surface enlargement were found in fish exposed to 20 °C for 32 days, as evidenced by the similar measurements of the primary and secondary lamellae compared to those at 9 °C. However, it is important to note that the complex three-dimensional structure of the gills may not be fully captured through two-dimensional analysis [31]. The literature on the effect of temperature on gill epithelial height varies. In a grouper hybrid, gill secondary lamella thickness increased within suboptimal temperature ranges, which aligns with the present findings [8]. Conversely, in Hoven’s carp, *Leptobarbus hoevenii*, a temperature increase of 4 °C above the optimal range decreased gill epithelial thickness [6]. Several studies have shown an increase in gill epithelial height following exposure to toxicants and environmental pollutants [32,33,34], suggesting that such changes may serve as a general protective mechanism under suboptimal environmental conditions.

For optimal gas exchange, gills have a large surface and a low epithelial thickness (i.e., short diffusion distance) [35,36,37]. These structural characteristics result also in high ion fluxes (in freshwater teleosts, the loss of salts via diffusion) and water fluxes (influx via osmosis) and therefore in considerable osmoregulatory costs [35,36,37]. Thus, the thickness and surface area of the gill epithelium represent a compromise between conflicting demands [35,36,37]. Increasing the surface area and reducing the epithelial height enhance gaseous exchange and water/ion fluxes but also elevate osmoregulatory costs, whereas the reverse adaptations decrease gaseous exchange efficiency but reduce osmoregulatory costs [35,37]. Following this theory, the increase in gill epithelial thickness could be considered an adaptation to reduce the energetic costs associated with osmoregulation. This hypothesis is supported by the observed decrease in gill H^+^-ATPase and Na^+^/K^+^-ATPase in rainbow trout exposed to 20 °C for 32 days. These enzymes function in series as membrane ion-pumps [38]. H^+^-ATPase is located apically and Na^+^/K^+^-ATPase is located basolaterally in the gill epithelium and they act together in series as membrane ion-pumps [38]. The H^+^-ATPase transports protons to the surrounding water and creates the electro-chemical gradient that enables Na^+^ to diffuse in through apical Na^+^ channels. Basolaterally, Na^+^ is pumped into the blood by Na^+^/K^+^-ATPase [38]. Alterations in enzyme activity did not affect the ion composition of blood plasma, which remained similar between rainbow trout acclimated to 9 °C and those long-term exposed to 20 °C [15]. The unfavorable conditions for gaseous exchange may be compensated by the observed increase in carbonic anhydrase activity, facilitating CO_2_ transport and excretion [39].

Another compensatory mechanism could be the previously described reduction in the erythrocyte size of rainbow trout in response to elevated temperatures, which amounted to 7% in cell volume and cell surface and 15% in nucleus volume, and which might facilitate gaseous exchange [15]. Similar changes in erythrocyte size were observed in the experiment described here (unpublished data). Compensatory mechanisms might also concern the rate of blood circulation. Possible adaptations for changes in the rate of blood circulation might include flow redistribution within the secondary lamellae, low shunting between the respiratory and ionoregulatory areas, and the enhancement of blood flow in the distal parts of the gill filaments [3,40]. No data are available on the rate of blood flow in rainbow trout gills in relation to temperature. This topic must be investigated in future studies. In the present study, the number of capillaries in the secondary lamellae revealed no differences between the fish kept at 9 °C and those kept at 20 °C, indicating no changes in the density of the capillary network.

The activities of Na^+^/K^+^-ATPase, H^+^-ATPase, and carbonic anhydrase are also linked to acid–base regulation, primarily modulating Cl^−^/HCO^3−^ and Na^+^/H^+^ exchanges at the gill to adjust blood plasma HCO^3−^ concentration [34]. Changes in enzyme activity may also indicate a shift in acid–base regulation at elevated temperatures [35,41]. Elevated body temperatures in ectothermic vertebrates can lead to increases in arterial _P_CO_2_ and a decline in the resting arterial pH [41,42].

The functions described for the gills require high levels of energy [43,44]. According to the generally accepted concept today, the specialized cells of the gills do not store energy in the form of glycogen or lipids; instead, energy is transported to the gills via blood flow in the form of glucose [45,46]. This is supported by data indicating that blood and gill glucose levels are in equilibrium [45,46]. Therefore, the energy required by the gills is mainly delivered through glycolysis, while fatty acids and amino acids play no roles as energy substrates [43,44,45,46]. Glucose derives from the glycogen storage depots of the liver [45,46]. A previous study on rainbow trout blood composition after exposure to 20 °C for 32 days indicated that blood glucose levels do not change in response to elevated temperatures [15]. Consequently, it might be suggested that gill energy resources are not restricted at 20 °C. Pyruvate kinase, a rate-controlling enzyme of glycolysis (catalyzing the transfer of a phosphate group from phosphoenolpyruvate to adenosine diphosphate), showed lower activity at 20 °C compared to 9 °C, while lactate dehydrogenase, responsible for converting pyruvate to lactate for energy production under low oxygen conditions, increased. This suggests a decrease in the glycolytic rate and a greater reliance on anaerobic pathways at 20 °C compared to 9 °C. Malate dehydrogenase, a key enzyme of the citric acid cycle catalyzing the oxidation of malate to oxaloacetate, remained constant, indicating the consistent efficiency of this pathway in the gills of rainbow trout in response to elevated temperatures. However, a more detailed screening is required to fully understand these changes. Generally, glycolysis is a metabolic pathway independent of oxygen, with the exception of its last step [47]. In the last step, pyruvate enters the citric acid cycle and undergoes oxidative phosphorylation under aerobic conditions. Under anaerobic conditions, pyruvate converts to lactate, catalyzed by lactate dehydrogenase, with an efficiency of energy production that is 95% lower than that for aerobic glycolysis [47]. The citric acid cycle cannot occur in the absence of oxygen because there is no possibility to regenerate the NAD^+^ used during this process [47].

The present study did not observe any histopathological changes in the gills (e.g., cell or tissue necrosis) affecting their function, indicating the adaptability of rainbow trout to the tested temperature regime. However, previous studies on various teleost species have reported different histopathological changes in gills in response to elevated temperatures [6,8,11,12,13].

## 5. Conclusions

The study revealed significant alterations in both gill structure and metabolism in rainbow trout exposed to 20 °C for 32 days, suggesting an adaptive response to elevated temperatures. These alterations included an increase in the thickness of the gill epithelium, a reduction in the activity of gill H^+^-ATPase and Na^+^/K^+^-ATPase, and a rise in carbonic anhydrase activity. These changes likely impact the processes of gaseous exchange and osmoregulation. Additionally, the enzymes involved in gill energy metabolism underwent changes. Conversely, there were no signs of gill surface enlargement in fish exposed to 20 °C for 32 days, nor were there any histopathological changes observed in the gills as a result of elevated temperature exposure.

## Figures and Tables

**Table 1:** Changes in morphometric parameters of the gill of the rainbow trout exposed to 20 °C for 32 d in comparison to fish acclimated to 9 °C. Data are mean ± standard deviation (*n* = 20). Data superscripted by different letters are significantly different. Student *t*-test, *p* < 0.005.

Morphometric Parameter	9 °C	20 °C
**Parameters with significant differences**		
Width of secondary lamellae, µm	13.1 ± 1.4 ^a^	18.0 ± 2.1 ^b^
Mean epithelial thickness, µm	2.5 ± 0.3 ^a^	3.5 ± 0.3 ^b^
**Parameters without differences**		
Length of primary lamellae, µm	4534 ± 390 ^a^	4546 ± 364 ^a^
Distance between primary lamellae, µm	308 ± 16 ^a^	314 ± 17 ^a^
Length of secondary lamellae, mm	239 ± 25 ^a^	244 ± 39 ^a^
Distance between secondary lamellae, µm	30.0 ± 4.4 ^a^	27.7 ± 5.6 ^a^
No. of mucus cells/mm	6.9 ± 2.39 ^a^	8.1 ± 3.1 ^a^
No. of chloride cells/mm	8.9 ± 3.4 ^a^	10.6 ± 3.1 ^a^
No. of capillaries/mm	69.5 ± 11.6 ^a^	66.2 ± 13.8 ^a^

**Table 2:** Changes in gill enzyme activities of rainbow trout exposed to 20 °C for 32 d in comparison to fish acclimated to 9 °C. Data are mean standard deviation (*n* = 20), those superscripted by different letters are significantly different. Student *t*-test, *p* < 0.005.

Enzymes (µmol/min/g Protein)	9 °C	20 °C
**Enzymes with significant differences**		
Lactate dehydrogenase	159.7 ± 33.4 ^a^	210.9 ± 47.8 ^b^
Pyruvate kinase	1.27 ± 0.38 ^a^	0.63 ± 0.61 ^b^
Na^+^/K^+^ ATPase	2.42 ± 0.90 ^a^	1.73 ± 0.89 ^b^
H^+^ ATPase	1.61 ± 0.66 ^a^	1.11 ± 0.45 ^b^
Carbonic anhydrase	3.60 ± 1.19 ^a^	5.63 ± 1.25 ^b^
**Enzymes without differences**		
Malate dehydrogenase	9.78 ± 3.86 ^a^	8.61 ± 2.79 ^a^

## Data Availability

The data presented in this study are available on request from the corresponding author. The data are not publicly available due to [the policy of the Federal Agency for Water Management].
